# Prevalence of Thrombocytopenia Among Pregnant Women at Rabia Balkhi Hospital in Afghanistan: A Cross-Sectional Study

**DOI:** 10.7759/cureus.117961

**Published:** 2026-10-15

**Authors:** Masooda Ahmadzai, Ahmad Saber Ahmadzai

**Affiliations:** 1 General Internal Medicine, Rabia Balkhi Hospital, Kabul, AFG; 2 Computer Science, Independent Researcher, Kabul, AFG

**Keywords:** afghanistan, hypertensive disorders of pregnancy, platelet count, pregnancy, thrombocytopenia

## Abstract

Background and objective: Thrombocytopenia is common during pregnancy, but published evidence from Afghanistan is limited. This study aimed to estimate the prevalence of documented thrombocytopenia among pregnant women admitted to selected wards at Rabia Balkhi Hospital, Kabul, and, among affected women, to describe severity, trimester distribution, maternal age, platelet count, and recorded diagnostic categories.

Materials and methods: We conducted a retrospective cross-sectional study of inpatient registers and medical records from the general internal medicine and obstetric emergency wards between August 23, 2025, and March 20, 2026. The registers contained 1,760 unique pregnant women. Medical records of 138 women identified as having thrombocytopenia were reviewed, and all 138 women had platelet counts below 150 × 10⁹/L. Severity was classified as mild (100-149 × 10⁹/L), moderate (50-99 × 10⁹/L), or severe (<50 × 10⁹/L). Data were summarized descriptively using SPSS Statistics version 32.0 (IBM Corp. Released 2026. IBM SPSS Statistics for Windows, Version 32.0. Armonk, NY).

Results: The documented prevalence of thrombocytopenia was 7.84% (138/1,760; 95% confidence interval: 6.67-9.19%). Mild, moderate, and severe thrombocytopenia occurred in 46 (33.3%), 64 (46.4%), and 28 (20.3%) women, respectively. Most affected women were in the third trimester (114; 82.6%). Hypertensive disorders of pregnancy constituted the largest recorded diagnostic category (79; 57.2%), including hemolysis, elevated liver enzymes, and low platelet count (HELLP) syndrome (35 cases), preeclampsia (28 cases), and eclampsia (16 cases). Gestational thrombocytopenia accounted for 24 cases (17.4%), immune thrombocytopenia for nine (6.5%), and other or associated conditions for 26 (18.8%).

Conclusions: Documented thrombocytopenia affected 7.84% of pregnant women registered in the selected wards. Moderate thrombocytopenia was the most frequent severity category, and hypertensive disorders of pregnancy constituted the largest recorded diagnostic category. The findings apply to this selected tertiary hospital population and should not be generalized to routine antenatal populations.

## Introduction

Thrombocytopenia, commonly defined as a platelet count below 150 × 10⁹/L, is a frequent hematologic finding during pregnancy [1-2]. Platelet counts tend to decline modestly as gestation progresses [3]. Thrombocytopenia may also indicate a clinically significant maternal or pregnancy-related disorder. The differential diagnosis includes gestational thrombocytopenia, hypertensive disorders of pregnancy, immune thrombocytopenia (ITP), infections, thrombotic microangiopathies, disseminated intravascular coagulation, and other systemic conditions [1,2,4-8]. Distinguishing a benign gestational change from a pathological process requires consideration of the platelet count, gestational age, clinical context, and underlying diagnosis [1-8].

The reported prevalence of thrombocytopenia in pregnancy varies across populations and clinical settings. Reviews generally estimate a frequency of about 5-10% [2], while individual studies report both lower and higher values. Studies from India and Ethiopia report prevalence values of 8.8% to 14.5% [9-11]. A systematic review of African studies estimated a pooled prevalence of 10.23% [12], and a 2025 Ethiopian meta-analysis reported a pooled prevalence of 10.7% [13]. Differences in sampling, trimester distribution, case definitions, referral patterns, and clinical setting may account for some of this variation [9-13].

Published evidence on thrombocytopenia among pregnant women in Afghanistan is limited [14]. This study therefore aimed to estimate the prevalence of documented thrombocytopenia among pregnant women admitted to the general internal medicine and obstetric emergency wards of Rabia Balkhi Hospital and, among affected women, to describe thrombocytopenia severity, trimester distribution, maternal age, platelet count, and recorded diagnostic categories.

## Materials and methods

Study design and setting

We conducted a retrospective cross-sectional study of existing inpatient registers and medical records at Rabia Balkhi Hospital in Kabul, Afghanistan. The hospital is a tertiary obstetric referral center. The study population comprised pregnant women admitted to the general internal medicine and obstetric emergency wards from August 23, 2025, to March 20, 2026. The study design, setting, selected wards, and study period were consistent with the approved research proposal. Data abstraction began after ethical approval and hospital authorization were obtained.

Study population and eligibility

The inpatient registers of the selected wards contained 1,760 unique pregnant women admitted during the study period. The registers were screened to identify women with documented thrombocytopenia, after which their corresponding medical records were retrieved. All 138 women identified as having thrombocytopenia had a platelet count below 150 × 10⁹/L, and all were included in the individual-level analysis. No identified case was excluded. The remaining 1,622 women were not identified in the registers as having thrombocytopenia and remained in the prevalence denominator; their individual medical records were not reviewed. Each woman was counted once, and duplicate records were not included.

Data source and variables

Data for the 138 women with documented thrombocytopenia were retrospectively extracted from their medical records. The variables collected were maternal age, trimester of pregnancy, platelet count, thrombocytopenia severity, and the diagnosis recorded by the treating physician. Hypertensive disorders of pregnancy included HELLP syndrome, preeclampsia, and eclampsia. Gestational thrombocytopenia and ITP were analyzed as separate categories. Infection-associated diagnoses, megaloblastic anemia, disseminated intravascular coagulation, and acute fatty liver of pregnancy were grouped as other or associated conditions.

One diagnostic category and one platelet count were recorded for each woman. If an initial platelet count was considered questionable, the confirmed repeat result documented in the medical record was used. Otherwise, the documented platelet count available for that case was entered. We did not apply a standardized rule requiring selection of either the first or the lowest measurement. Details of serial platelet measurements and peripheral-blood-smear confirmation were not collected separately. The data-abstraction form also did not capture information on detailed etiological investigations, bleeding events, treatment, platelet transfusions, mode of delivery, neuraxial anesthesia, or maternal, fetal, and neonatal outcomes. Therefore, the investigators did not reassess the diagnoses using standardized diagnostic criteria; instead, the diagnoses were analyzed as documented by the treating physicians.

Operational definitions

Thrombocytopenia was defined as a platelet count below 150 × 10⁹/L. Severity was classified according to the prespecified study definition as mild (100-149 × 10⁹/L), moderate (50-99 × 10⁹/L), or severe (<50 × 10⁹/L). Platelet counts are reported in × 10⁹/L and age in years.

Statistical analysis

Data were analyzed using SPSS Statistics version 32.0 (IBM Corp. Released 2026. IBM SPSS Statistics for Windows, Version 32.0. Armonk, NY). Documented prevalence was calculated by dividing the number of women with register-identified thrombocytopenia confirmed through medical record review by the total number of unique pregnant women in the selected ward registers and multiplying by 100. A two-sided 95% Wilson confidence interval was calculated for the prevalence estimate. Categorical variables were summarized as frequencies and percentages; maternal age as the mean and standard deviation; and platelet count as the median, lower and upper quartiles, and range. Crosstabulations were used to describe severity across recorded diagnostic categories. The analysis was descriptive, and inferential statistics were not used to support the conclusions.

Ethical considerations

The Scientific Committee of the General Directorate of Postgraduate Medical Education, Ministry of Public Health, Afghanistan, granted scientific approval. The Institutional Review Board of the Afghanistan National Public Health Institute granted ethical approval (IRB Code No. A-6-26-514; June 21, 2026). Rabia Balkhi Hospital authorized access to the medical records. The study used de-identified records and involved no patient contact; the IRB therefore waived informed consent. The study was conducted in accordance with the Declaration of Helsinki.

## Results

Participant identification and documented prevalence

The inpatient registers contained 1,760 unique pregnant women admitted to the selected wards during the study period. Thrombocytopenia was documented in 138 women, and a review of their medical records confirmed a platelet count below 150 × 10⁹/L in all cases. No cases were excluded, and no woman was included twice. The documented prevalence of thrombocytopenia was 7.84% (138/1,760; 95% confidence interval: 6.67-9.19%). Figure 1 shows participant identification.

**Figure 1:**
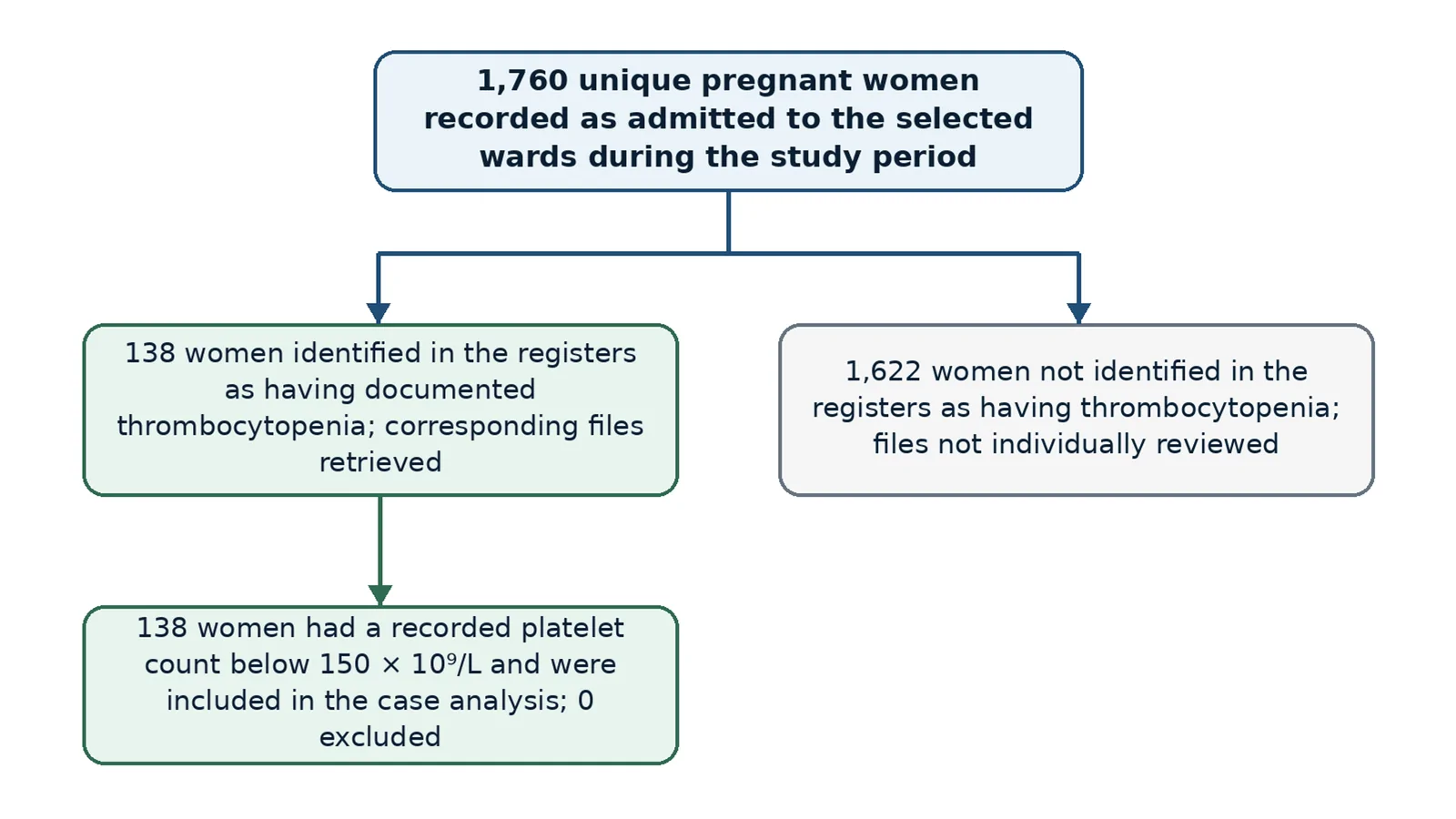
Identification of women with documented thrombocytopenia

Each woman was counted once. The 1,622 women not identified in the registers as having thrombocytopenia remained in the prevalence denominator, but their medical records were not individually reviewed.

Characteristics of affected women

Data on maternal age, platelet count, severity, trimester, and recorded diagnostic category were complete for all 138 affected women. The mean age was 30.67 ± 5.54 years (range, 18-43 years). The median platelet count was 83.50 × 10⁹/L (lower quartile, 56.75 × 10⁹/L; upper quartile, 116.00 × 10⁹/L; range, 8-145 × 10⁹/L). Most affected women were in the third trimester (114; 82.6%), followed by the second trimester (19; 13.8%) and the first trimester (five; 3.6%) (Table 1).

**Table 1:** Selected characteristics of women with documented thrombocytopenia (n = 138) Q1: lower quartile, Q3: upper quartile, SD: standard deviation

Characteristic	Result
Age, years, mean ± SD	30.67 ± 5.54
Age range, years	18-43
Platelet count, × 10⁹/L, median (Q1-Q3)	83.50 (56.75-116.00)
Platelet-count range, × 10⁹/L	8-145
First trimester, n (%)	5 (3.6)
Second trimester, n (%)	19 (13.8)
Third trimester, n (%)	114 (82.6)

Severity and recorded diagnostic categories

Moderate thrombocytopenia was the most frequent severity category, occurring in 64 women (46.4%), followed by mild thrombocytopenia in 46 (33.3%) and severe thrombocytopenia in 28 (20.3%). Moderate and severe thrombocytopenia together accounted for 92 of 138 women (66.7%).

Hypertensive disorders of pregnancy constituted the largest recorded diagnostic category, accounting for 79 women (57.2%): HELLP syndrome in 35, preeclampsia in 28, and eclampsia in 16. Gestational thrombocytopenia was recorded in 24 women (17.4%), ITP in nine (6.5%), and other or associated conditions in 26 (18.8%). The other or associated conditions category comprised 14 infection-associated diagnoses, including malaria; five cases of megaloblastic anemia; four cases of disseminated intravascular coagulation; and three cases of acute fatty liver of pregnancy (Table 2).

**Table 2:** Severity and detailed recorded diagnostic categories (n = 138) HELLP syndrome, preeclampsia, and eclampsia together constituted the hypertensive-disorder group (n = 79). HELLP: hemolysis, elevated liver enzymes, and low platelet count, ITP: immune thrombocytopenia

Measure	Category	n	%
Severity	Mild	46	33.3
Severity	Moderate	64	46.4
Severity	Severe	28	20.3
Recorded diagnosis	HELLP syndrome	35	25.4
Recorded diagnosis	Preeclampsia	28	20.3
Recorded diagnosis	Eclampsia	16	11.6
Recorded diagnosis	Gestational thrombocytopenia	24	17.4
Recorded diagnosis	ITP	9	6.5
Recorded diagnosis	Infection-associated, including malaria	14	10.1
Recorded diagnosis	Megaloblastic anemia	5	3.6
Recorded diagnosis	Disseminated intravascular coagulation	4	2.9
Recorded diagnosis	Acute fatty liver of pregnancy	3	2.2

Descriptive distribution of severity by recorded diagnostic category

All 24 women classified as having gestational thrombocytopenia had mild thrombocytopenia. Among the 79 women with recorded hypertensive disorders of pregnancy, 48 (60.8%) had moderate thrombocytopenia, 17 (21.5%) had mild thrombocytopenia, and 14 (17.7%) had severe thrombocytopenia. Among the nine women with ITP, seven (77.8%) had severe thrombocytopenia, and two (22.2%) had moderate thrombocytopenia. In the other or associated conditions category, 14 of 26 women (53.8%) had moderate thrombocytopenia, seven (26.9%) had severe thrombocytopenia, and five (19.2%) had mild thrombocytopenia (Table 3). These distributions are descriptive and do not, by themselves, establish an association between recorded diagnostic category and thrombocytopenia severity.

**Table 3:** Descriptive distribution of thrombocytopenia severity by recorded diagnostic category Percentages are calculated within each recorded diagnostic category. The table is descriptive and is not interpreted as establishing an association.

Recorded diagnostic category	Mild n (%)	Moderate n (%)	Severe n (%)	Total n
Gestational thrombocytopenia	24 (100.0)	0 (0.0)	0 (0.0)	24
Hypertensive disorders	17 (21.5)	48 (60.8)	14 (17.7)	79
ITP	0 (0.0)	2 (22.2)	7 (77.8)	9
Other or associated conditions	5 (19.2)	14 (53.8)	7 (26.9)	26
Total	46 (33.3)	64 (46.4)	28 (20.3)	138

## Discussion

In this retrospective study, documented thrombocytopenia was identified in 7.84% of pregnant women admitted to the selected wards of Rabia Balkhi Hospital. Moderate thrombocytopenia was the most frequent severity category; most affected women were in the third trimester, and hypertensive disorders of pregnancy constituted the largest recorded diagnostic category. These findings describe women admitted to the general internal medicine and obstetric emergency wards of a tertiary referral hospital rather than an unselected antenatal population.

The documented prevalence of 7.84% is close to the 8.8% reported by Nisha et al. in India and Asrie et al. in northwest Ethiopia [9,10]. It is lower than the 14.5% reported by Haile et al. in southwest Ethiopia [11], the pooled African estimate of 10.23% reported by Getawa et al. [12], and the pooled Ethiopian estimate of 10.7% reported in 2025 [13]. It is higher than the 5.6% reported by Sridhar et al. in Karnataka [15]. Direct comparisons should be interpreted cautiously because these studies differed in setting, eligibility criteria, trimester distribution, case definitions, and referral patterns. In the present study, case identification was based on documented thrombocytopenia in the inpatient registers of two hospital wards.

The severity distribution also differed from several published reports. Moderate and severe thrombocytopenia together accounted for 66.7% of affected women in this study. Haile et al. reported mild thrombocytopenia in 77.0% and severe thrombocytopenia in 6.6% of affected women [11], while the African meta-analysis also found mild thrombocytopenia to predominate [12]. Sridhar et al. reported moderate thrombocytopenia in 67.2% and severe thrombocytopenia in 31.4% [15]. The relatively high proportion of moderate or severe thrombocytopenia in the present study may reflect the case mix of medical and obstetric emergency admissions; however, the descriptive study design does not permit this explanation to be tested.

Women in the third trimester accounted for 82.6% of thrombocytopenic cases. Platelet counts tend to decline as pregnancy progresses, and gestational thrombocytopenia is commonly recognized later in gestation [3,8]. The African meta-analysis also reported the highest proportion of thrombocytopenia during the third trimester [12]. The figure of 82.6% represents the proportion of affected women in the third trimester rather than a trimester-specific prevalence estimate. Denominator data for all registered women in each trimester were unavailable; therefore, we could not calculate or compare prevalence across trimesters.

Gestational thrombocytopenia is generally the most frequent cause of thrombocytopenia in pregnancy and is usually mild [2,4,8]. It accounted for 64.2% of affected women in Nisha et al.'s study [9], 39.3% in Sridhar et al.'s Karnataka study [15], and 74.7% in the Northern Pakistan series reported by Ellahi et al. [16]. In this study, hypertensive disorders of pregnancy were recorded in 57.2% of affected women, including 35 with HELLP syndrome, 28 with preeclampsia, and 16 with eclampsia. Preeclampsia and HELLP syndrome may be accompanied by thrombocytopenia through endothelial injury, platelet activation, and increased platelet consumption [1,2,4-7]. The tertiary referral setting and restriction to the general internal medicine and obstetric emergency wards may have increased the representation of complicated pregnancies. This may explain the observed pattern; however, the study did not collect the variables necessary to evaluate causal mechanisms or the potential contributions of autoimmune disease, antiphospholipid antibody syndrome, or thrombotic microangiopathy. The results therefore do not establish hypertensive disorders of pregnancy as the most common cause of thrombocytopenia among all pregnant women in Kabul or Afghanistan.

The distribution of thrombocytopenia severity across recorded diagnostic categories was clinically plausible. All women classified as having gestational thrombocytopenia had mild thrombocytopenia, seven of the nine women with ITP had severe thrombocytopenia, and most women in the hypertensive-disorder group had moderate thrombocytopenia. Clinical reviews describe gestational thrombocytopenia as typically mild and indicate that ITP and pregnancy-related hypertensive disorders may be associated with lower platelet counts [2,4-8]. However, this study relied on diagnostic categories documented by treating physicians and did not independently adjudicate them using a standardized etiological assessment. Therefore, these categories should be interpreted as recorded diagnoses rather than independently confirmed etiological diagnoses.

This study has several strengths. It provides local data from a setting in which published evidence is limited [14], uses a prespecified definition of thrombocytopenia and consistent severity thresholds, and includes all registered pregnant women admitted to the selected wards during the study period in the prevalence denominator.

Limitations

This study has several limitations. Its retrospective, single-center design relied on routinely collected medical records, and the medical records of women not identified as having thrombocytopenia were not individually reviewed. Consequently, unrecorded cases may have been missed, limiting the generalizability of the findings. Diagnostic categories were based on treating physicians’ documentation, and the analytical dataset did not include detailed information on etiological assessments, systematic platelet-count confirmation, treatment, or maternal, fetal, and neonatal outcomes. The relatively small number of affected women also limited the interpretation of subgroup findings. Accordingly, the findings describe documented cases in the selected wards and should not be interpreted as population-wide estimates or causal associations. Prospective multicenter studies using standardized diagnostic assessments and maternal, fetal, and neonatal follow-up are needed to confirm these findings.

## Conclusions

Among pregnant women admitted to the general internal medicine and obstetric emergency wards of Rabia Balkhi Hospital, a proportion had documented thrombocytopenia. Moderate thrombocytopenia was the most frequent severity category; most affected women were in the third trimester, and hypertensive disorders of pregnancy constituted the largest recorded diagnostic category. Because the study population was restricted to pregnant women admitted to selected wards, and individual medical records were reviewed only for women identified as having thrombocytopenia, these findings should not be extrapolated to routine antenatal populations in Kabul or Afghanistan. Multicenter studies with individual-level data for the full denominator and standardized diagnostic assessments would help determine whether similar patterns are present in other pregnant populations in Afghanistan.
